# Pore Orientation Affects the Compression Resistance of Unidirectional Porous Beta-Tricalcium Phosphate Wedges in Opening-Wedge High Tibial Osteotomy

**DOI:** 10.7759/cureus.115507

**Published:** 2026-08-31

**Authors:** Shinya Kawahara, Takeshi Shimoto, Satoshi Hamai, Yukio Akasaki, Taishi Sato, Yasuharu Nakashima

**Affiliations:** 1 Department of Orthopaedic Surgery, Graduate School of Medical Sciences, Kyushu University, Fukuoka, JPN; 2 Department of Information and System Engineering, Faculty of Information Engineering, Fukuoka Institute of Technology, Fukuoka, JPN; 3 Department of Orthopaedic Surgery, Kyushu University Hospital, Fukuoka, JPN

**Keywords:** artificial bone, beta-tricalcium phosphate, biomechanics, bone substitute, compression testing, high tibial osteotomy, opening-wedge osteotomy, pore orientation

## Abstract

Background: Unidirectional porous beta-tricalcium phosphate is used as a synthetic bone substitute in opening-wedge high tibial osteotomy. Because this material has anisotropic mechanical properties, the orientation of its interconnected pores may influence initial support within the osteotomy gap. This study evaluated whether pore orientation affects the compression resistance of unidirectional porous beta-tricalcium phosphate wedges in an opening-wedge high tibial osteotomy-like loading model.

Materials and methods: This in vitro biomechanical study evaluated two wedge sizes: WA08, corresponding to an approximately 7.6-degree wedge, and WA14, corresponding to an approximately 14.3-degree wedge. For each size, three configurations were tested: the commercially preformed wedge with pores parallel to the medial and lateral faces, a processed block with pores perpendicular to the lower surface, and a processed block with pores perpendicular to the upper surface. Ten specimens were tested per configuration. Monotonic compression was applied using a hinged fixture that reproduced the geometry of an opening-wedge high tibial osteotomy gap. Load-displacement and nominal contact pressure-displacement curves were compared by pairwise comparisons at each 0.01 mm displacement increment.

Results: In WA08, the commercially preformed configuration showed greater resistance at larger displacements, with significant differences from the lower-surface and upper-surface perpendicular configurations beginning at 0.37 and 0.41 mm, respectively. In WA14, the commercially preformed and upper-surface perpendicular configurations showed higher load-displacement and nominal pressure-displacement responses than the lower-surface perpendicular configuration.

Conclusions: Pore orientation affected the initial compression behavior of unidirectional porous beta-tricalcium phosphate wedges in a simulated opening-wedge high tibial osteotomy gap. The commercially preformed configuration showed the most consistent resistance across wedge sizes, suggesting that pore orientation should be considered when selecting or shaping beta-tricalcium phosphate wedges for opening-wedge high tibial osteotomy.

## Introduction

Medial opening-wedge high tibial osteotomy is a joint-preserving procedure for medial compartment knee osteoarthritis and varus malalignment. The osteotomy gap may be left unfilled or filled with synthetic bone substitutes, depending on correction size, fixation stability, surgeon preference, and the expected contribution of the spacer to early support [1,2]. Synthetic bone substitutes used in this setting should provide mechanical support, fit the opened wedge geometry, permit biologic integration, and eventually allow replacement by host bone.

Beta-tricalcium phosphate is bioresorbable and osteoconductive and has been used clinically as a wedge spacer in opening-wedge high tibial osteotomy [3,4]. Affinos (Kuraray Co., Ltd., Tokyo, Japan) is a unidirectional porous beta-tricalcium phosphate material designed with longitudinal interconnected pores. Previous studies have reported bone remodeling and clinical use of this material, but the mechanical effect of pore orientation in a high tibial osteotomy-like wedge gap has not been fully clarified [4,5]. Because unidirectional pores create anisotropic load paths, the direction of the pores relative to the loaded wedge surfaces may influence initial compression resistance.

The objective of this study was to determine whether pore configuration affects the initial compression resistance of unidirectional porous beta-tricalcium phosphate wedges in an opening-wedge high tibial osteotomy-like loading model. The study assessed load-displacement and nominal contact pressure-displacement behavior in two clinically relevant wedge sizes. We hypothesized that pore configuration would affect the initial compression resistance of unidirectional porous beta-tricalcium phosphate wedges, and that the commercially preformed configuration would show higher and more consistent resistance across wedge sizes.

## Materials and methods

Specimens and configurations

Unidirectional porous beta-tricalcium phosphate wedges were evaluated in two sizes: WA08 (length, 30 mm; thickness, 10 mm; end heights, 4 and 8 mm; actual wedge angle, approximately 7.6 degrees; Figure 1A) and WA14 (length, 30 mm; thickness, 10 mm; end heights, 7.5 and 15 mm; actual wedge angle, approximately 14.3 degrees; Figure 1B). Ten specimens were tested for each configuration of each size.

**Figure 1:**
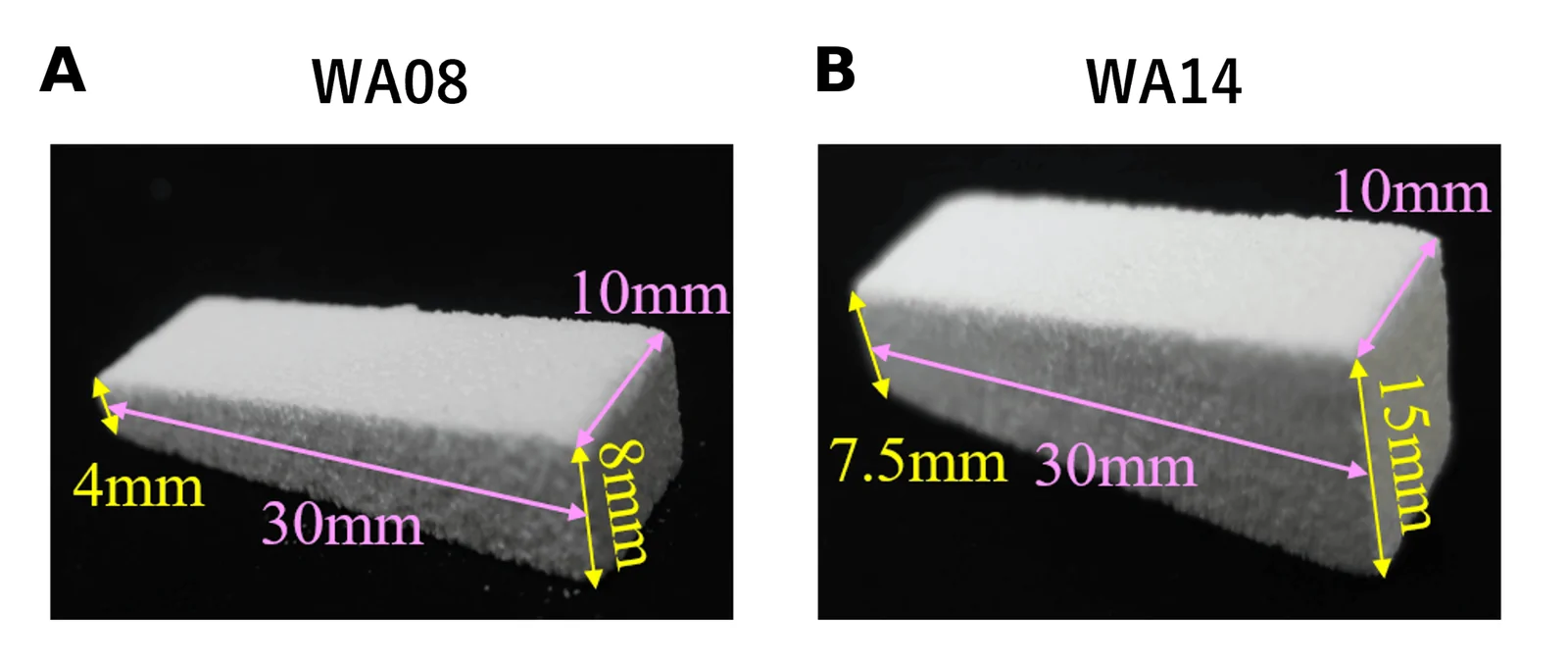
Specimen sizes used in this study. (A) WA08 measured 30 mm in length and 10 mm in thickness, with end heights of 4 and 8 mm. (B) WA14 measured 30 mm in length and 10 mm in thickness, with end heights of 7.5 and 15 mm.

Three pore configurations were tested for each size (Figure 2). Configuration A was the commercially preformed wedge, in which the interconnected pores were parallel to the medial and lateral faces (Figure 2A). Configuration B was shaped from a rectangular block so that the pore direction was perpendicular to the lower surface of the wedge (Figure 2B). Configuration C was shaped so that the pore direction was perpendicular to the upper surface of the wedge (Figure 2C). Configurations B and C were manufactured from rectangular unidirectional porous beta-tricalcium phosphate blocks by Kuraray Co., Ltd. using a cutting process similar to that used for commercially available wedge products. The specimens were prepared to match the target external dimensions of WA08 and WA14 as closely as possible. After processing, the external dimensions and pore orientation were checked before testing, and the processed surfaces maintained a surface texture similar to that of the commercial products. Preliminary testing confirmed reproducible load-displacement behavior among processed specimens.

**Figure 2:**
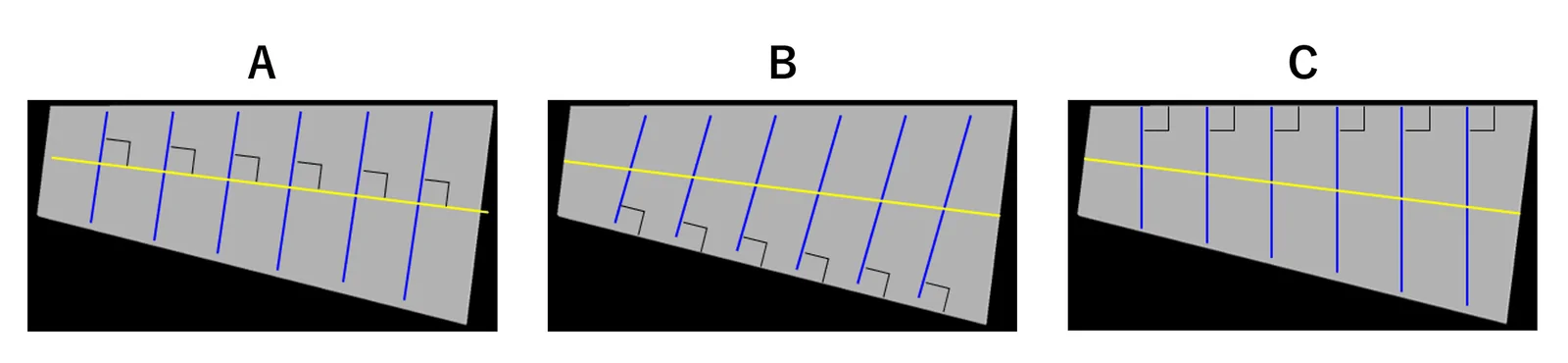
Classification of the unidirectional porous beta-tricalcium phosphate wedge configurations. (A) Configuration A was the commercially preformed wedge, with pores parallel to the medial and lateral faces. (B) Configuration B was shaped from a rectangular block, with pores perpendicular to the lower surface. (C) Configuration C was shaped from a rectangular block, with pores perpendicular to the upper surface.

Mechanical testing

A hinged fixture was designed to reproduce the geometry of an opening-wedge high tibial osteotomy gap. The testing machine is shown in Figure 3A, the specimen position within the fixture is shown in Figure 3B, and the schematic loading model is shown in Figure 3C. The center of each specimen was positioned 45 mm from the hinge axis, and the upper specimen surface was horizontal so that vertical load was applied directly above the specimen center. Sandpaper was attached to the contact surfaces, and a metal stopper was used to prevent specimen migration along the inclined lower surface.

**Figure 3:**
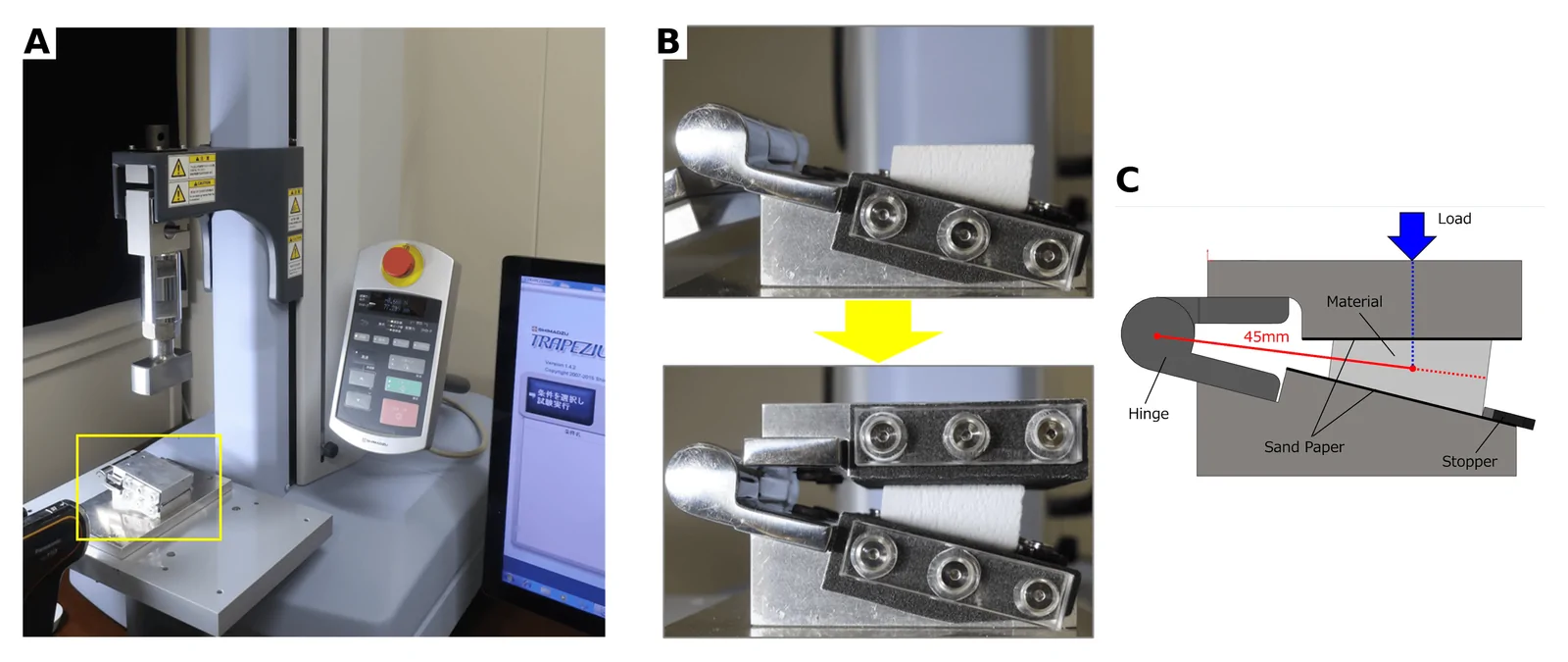
Compression testing setup. (A) Tabletop universal testing machine used for compression testing. (B) Specimen positioned within the hinged fixture. (C) Schematic of the opening-wedge high tibial osteotomy-like loading model. The specimen center was positioned 45 mm from the hinge axis, and vertical load was applied directly above the specimen center. Panel (C) created by the authors using Microsoft PowerPoint (Microsoft Corp., Redmond, WA, USA).

Compression testing was performed using a tabletop universal testing machine (EZ-LX; Shimadzu Corporation, Kyoto, Japan). The position at which a 5 N preload was reached was defined as zero displacement. Compression was applied at 0.5 mm/min and continued until the load reached 1,500 N.

Outcome variables and statistical analysis

The primary outcome was load-displacement behavior for each configuration. The secondary outcome was nominal contact pressure-displacement behavior. Nominal contact pressure was calculated by dividing the applied compression load by the nominal loaded cross-sectional area of each wedge. The loaded cross-sectional area was determined geometrically from the external dimensions of each specimen as the projected loaded area perpendicular to the vertical loading direction. Because configurations A, B, and C within each wedge size had the same external dimensions, the same nominal area was used for all three configurations within WA08 and within WA14, respectively. The nominal area was treated as constant during compression.

For each wedge size, data were summarized as mean curves with standard deviations from 10 independently tested specimens per configuration. Configurations A, B, and C were compared at each 0.01 mm displacement increment using pairwise two-sided unpaired t-tests. No formal adjustment for multiple comparisons across displacement increments was applied because these point-by-point analyses were intended to describe differences in curve behavior rather than to define independent clinical endpoints. Therefore, the results of these comparisons should be interpreted descriptively. Statistical analyses were performed using JMP Student Edition version 18.0 (JMP Statistical Discovery LLC, Cary, NC, USA), and a p-value < 0.05 was considered statistically significant.

## Results

Load-displacement behavior

Pore configuration changed load-displacement behavior in both wedge sizes (Figure 4). In WA08 (Figure 4A), configuration A showed greater resistance at larger displacements. Significant differences were observed between A and B from 0.37 mm and between A and C from 0.41 mm. Configurations B and C showed smaller and similar loads over the measured displacement range.

**Figure 4:**
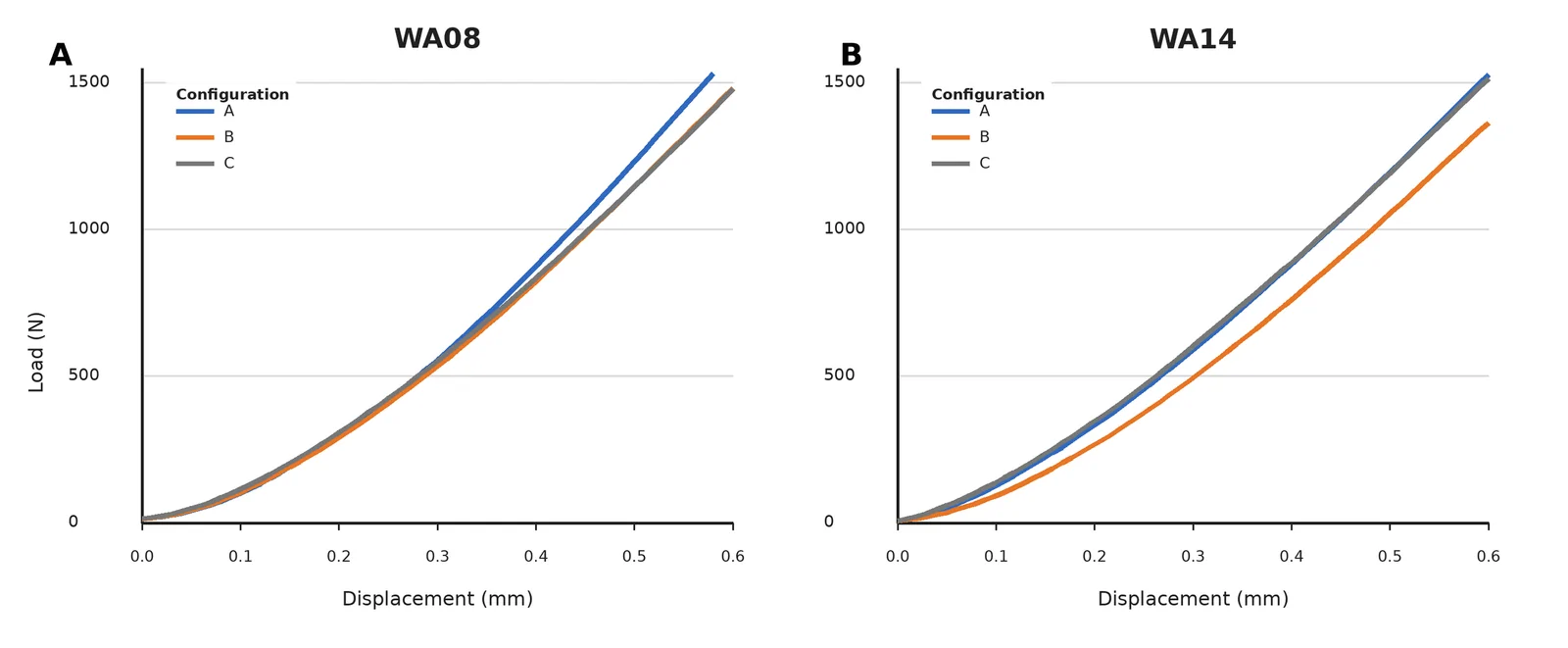
Mean load-displacement curves for each configuration (n = 10 per configuration). (A) WA08 wedges. Configuration A showed higher resistance than configurations B and C at larger displacements. (B) WA14 wedges. Configurations A and C showed higher resistance than configuration B.

In WA14 (Figure 4B), configurations A and C followed similar load-displacement trajectories, and both exceeded configuration B. Significant differences were observed between A and B from 0.02 mm and between C and B from 0.01 mm. No significant difference was detected between A and C over the measured displacement range.

Nominal contact pressure

The same general pattern was maintained after normalization to nominal contact pressure (Figure 5). In WA08 (Figure 5A), configuration A produced the highest nominal contact pressure across most of the displacement range. In WA14 (Figure 5B), configurations A and C remained higher than configuration B, indicating that the observed differences were not explained only by wedge size or loaded cross-sectional area.

**Figure 5:**
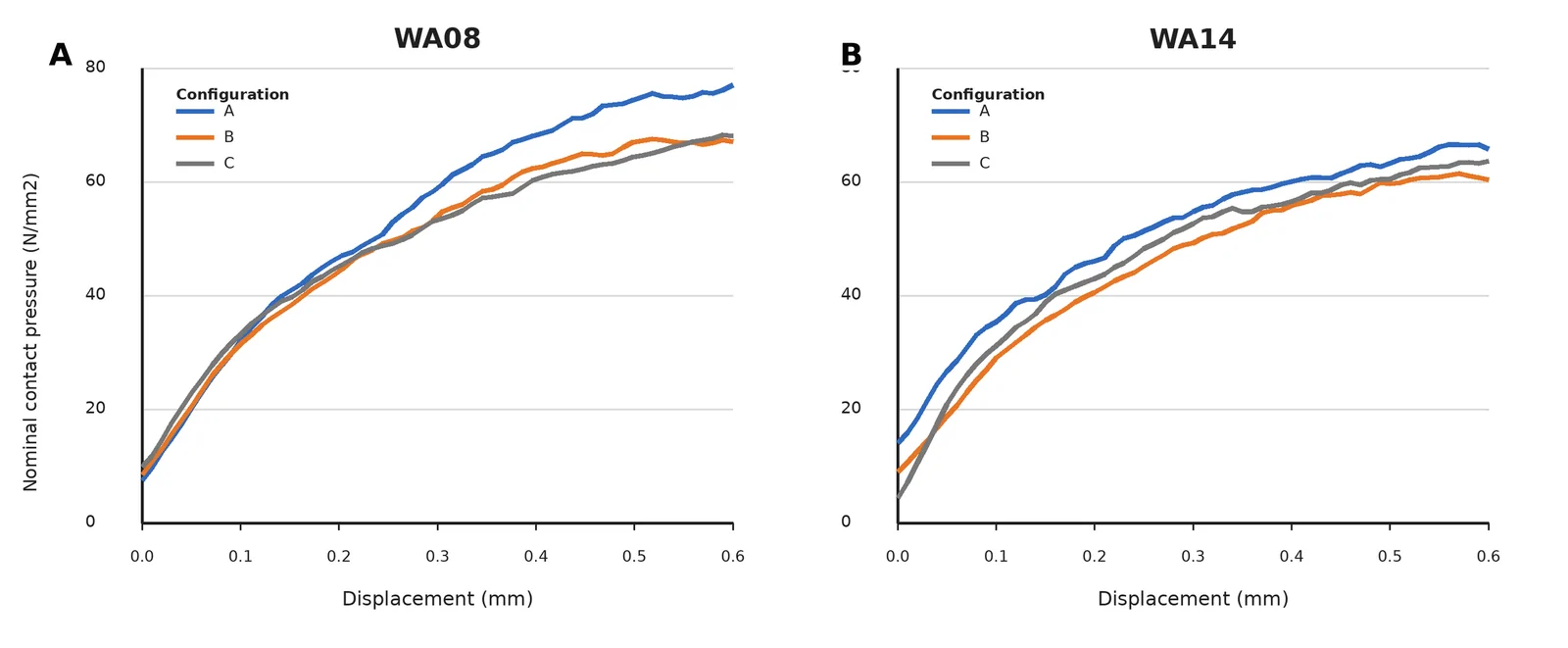
Nominal contact pressure-displacement curves after normalization by the loaded cross-sectional area. (A) WA08 wedges. Configuration A was highest across most of the displacement range. (B) WA14 wedges. Configurations A and C remained higher than configuration B.

## Discussion

The main finding of this in vitro study was that pore configuration affected the initial compression behavior of unidirectional porous beta-tricalcium phosphate wedges in an opening-wedge high tibial osteotomy-like loading model. Configuration A, the commercially preformed wedge, showed the most consistent mechanical behavior across the two wedge sizes, while configuration C also showed high resistance in the larger WA14 wedge. These findings add a material-orientation perspective to previous clinical discussions on whether and when an osteotomy gap filler should be used in opening-wedge high tibial osteotomy [1,2,6].

Unidirectional porous beta-tricalcium phosphate is mechanically anisotropic because longitudinally connected pores create different load paths parallel and perpendicular to the pore axis. This feature is clinically attractive because aligned interconnected pores may promote fluid infiltration and bone remodeling, as suggested by clinical and radiological studies of unidirectional porous beta-tricalcium phosphate in opening-wedge high tibial osteotomy [3,4]. Similar material concepts have also been applied to other metaphyseal bone defects, supporting the broader clinical interest in unidirectional porous beta-tricalcium phosphate as a bone substitute [5].

Normalization to nominal contact pressure did not reverse the results. Configuration A remained consistently in the upper range for both wedge sizes, whereas configuration C approached A in WA14. A possible explanation is that the commercially preformed wedge may distribute load more evenly between the upper and lower contact surfaces (Figure 6). In this model, the applied vertical load is transmitted from the upper contact surface; however, because the lower surface is inclined, part of the applied load may be redirected as shear along the lower surface. This would reduce the normal component acting through part of the wedge and may make configurations that receive load more uniformly across both surfaces mechanically advantageous. Configuration C, with pores perpendicular to the upper surface, may also resist the vertical load effectively, particularly in the larger WA14 wedge. This proposed load-distribution model should be interpreted as a hypothetical mechanical explanation, because contact pressure, shear force, and internal stress distribution were not directly measured in the present study. Therefore, the present findings support differences in initial compression resistance under this specific in vitro loading condition but do not demonstrate clinical effectiveness or in vivo superiority of any pore orientation.

**Figure 6:**
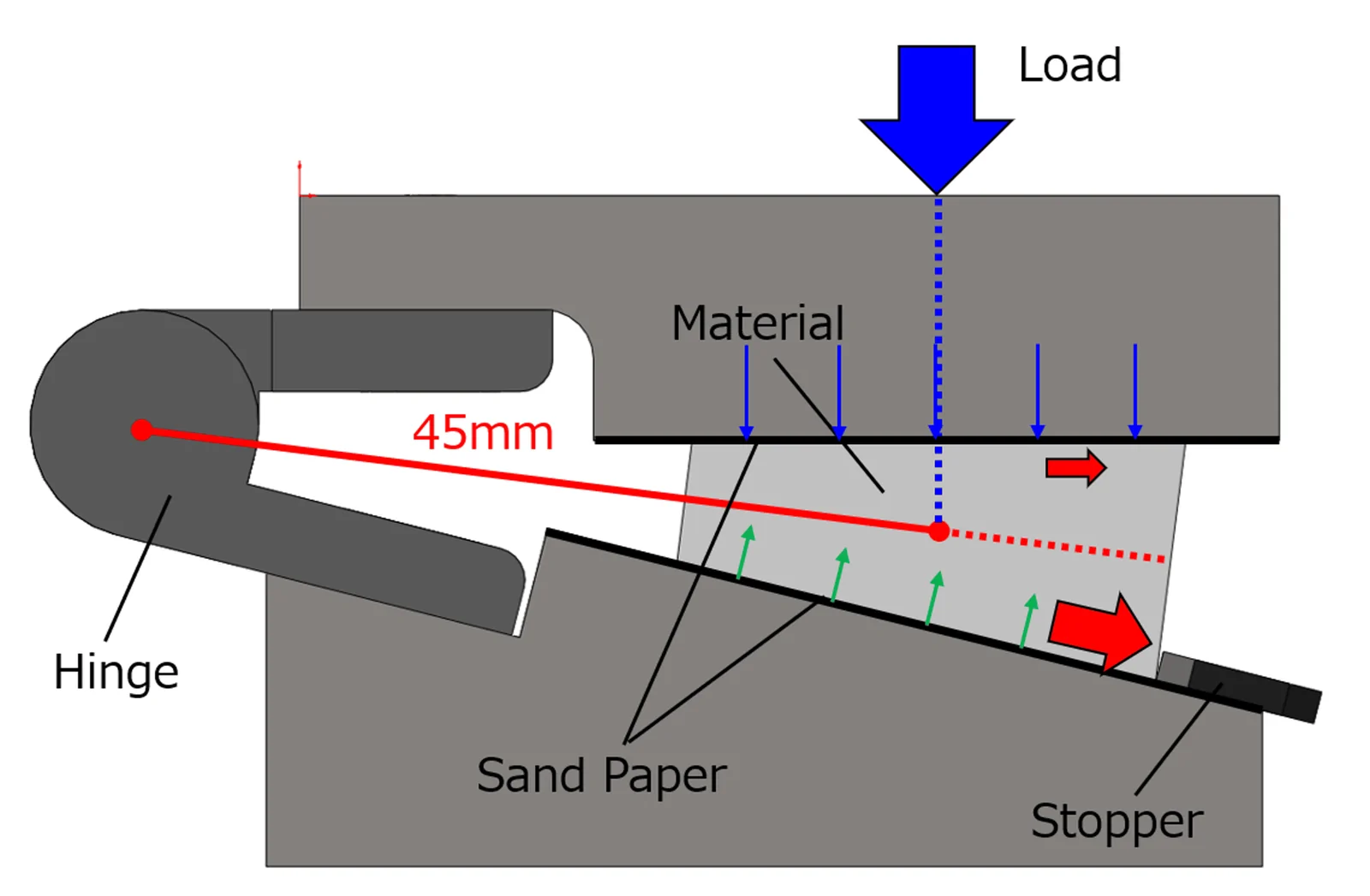
Proposed load distribution in the opening-wedge testing model. The blue arrows indicate load distribution from the upper contact surface. Because the lower surface is inclined, part of the applied load may be redirected as shear along the inclined surface, shown by red arrows, reducing the normal component acting through the specimen, shown by green arrows. This schematic represents a hypothetical mechanical explanation and was not validated by direct measurement of contact pressure, shear force, internal stress distribution, or finite element analysis. Figure created by the authors using Microsoft PowerPoint (Microsoft Corp., Redmond, WA, USA).

The clinical relevance is that, although a locking plate is the primary stabilizer in opening-wedge high tibial osteotomy, a wedge spacer may contribute to local support. Previous biomechanical studies have reported that synthetic wedges or graft materials increase the stability or static strength of medial opening-wedge high tibial osteotomy constructs compared with leaving the gap unfilled [7,8]. In addition, delayed gap healing has been associated with lateral hinge fracture, large opening gaps, and unfavorable osteotomy-line position [9,10]. These reports do not directly address pore orientation, but they support the importance of local mechanical support within the osteotomy gap, especially in mechanically demanding situations.

This study has several limitations. First, it was an in vitro monotonic compression test and did not reproduce bone quality, soft tissues, plate fixation, biologic remodeling, cyclic postoperative loading, or multiplanar physiological forces. Second, although sandpaper and a metal stopper were used to minimize specimen migration under controlled testing conditions, these boundary conditions created artificial frictional and constraint conditions. Mechanical behavior may differ when the wedges are surrounded by metaphyseal cancellous bone and incorporated within a plated osteotomy construct. Third, configuration A was a commercially preformed wedge, whereas configurations B and C were manufactured from rectangular blocks. Therefore, the comparisons may reflect not only pore orientation but also subtle differences in machining, surface geometry, dimensional tolerance, or processed surface integrity. Fourth, nominal contact pressure was calculated from the nominal loaded cross-sectional area and should not be interpreted as a direct measurement of true instantaneous contact area, local interface pressure, shear force, or internal stress distribution. Fifth, the proposed load-distribution model in Figure 6 is hypothetical and was not experimentally validated by direct measurement of contact pressure, shear force, internal stress distribution, or finite element analysis. Sixth, only two wedge sizes and three pore configurations were evaluated; therefore, the generalizability of the findings to other correction angles, wedge dimensions, implant geometries, loading directions, and physiological cyclic loading conditions remains uncertain. Finally, although pairwise comparisons were performed at each displacement increment, no formal adjustment for multiple comparisons across increments was applied. These analyses may increase the risk of type I error and should be interpreted descriptively as point-by-point comparisons of curve behavior rather than as independent clinical endpoints.

## Conclusions

Pore orientation affected the initial compression resistance of unidirectional porous beta-tricalcium phosphate wedges in a simulated opening-wedge high tibial osteotomy gap. The commercially preformed configuration showed the most consistent mechanical behavior across both wedge sizes, and the upper-surface perpendicular configuration also showed favorable resistance in the larger wedge.

These findings suggest that pore orientation should be considered when selecting or shaping beta-tricalcium phosphate wedges for opening-wedge high tibial osteotomy. Further studies incorporating physiological cyclic loading, plate fixation, bone models, comparisons with other bone substitutes, and clinical outcome data are needed to determine whether these in vitro mechanical differences translate into improved in vivo stability or clinical outcomes before definitive surgical recommendations can be made.
